# Retraction: miR-199a-3p knockdown inhibits dedifferentiated liposarcoma (DDLPS) cell viability and enhances apoptosis through targeting casein kinase-1 alpha (CK1α)

**DOI:** 10.1039/d1ra90070f

**Published:** 2021-02-01

**Authors:** Laura Fisher

**Affiliations:** Royal Society of Chemistry Thomas Graham House, Science Park, Milton Road Cambridge CB4 0WF UK advances-rsc@rsc.org

## Abstract

Retraction of ‘miR-199a-3p knockdown inhibits dedifferentiated liposarcoma (DDLPS) cell viability and enhances apoptosis through targeting casein kinase-1 alpha (CK1α)’ by Ye Cao *et al.*, *RSC Adv.*, 2019, **9**, 22755–22763, DOI: 10.1039/C9RA01491H.

The Royal Society of Chemistry hereby wholly retracts this *RSC Advances* article due to concerns with the reliability of the data. The images in the article were screened by an image integrity expert. The western blot panels contain very unusually regular-shaped bands. Furthermore, the blots and many other features of the article were found to very closely resemble blots and features from other papers, which is unexpected given that there are completely different author lists for these articles.

The authors were asked to provide the raw data for this article, but did not respond. Given the significance of the concerns about the validity of the data, and the lack of raw data, the findings in this paper are not reliable.

The authors were informed but have not responded to any correspondence regarding the retraction.

Signed: Laura Fisher, Executive Editor, *RSC Advances*

Date: 19^th^ January 2021

## Supplementary Material

